# The natural adaptive evolution of cancer: The metastatic ability of cancer cells

**DOI:** 10.17305/bjbms.2019.4565

**Published:** 2020-08

**Authors:** Gheorghe-Emilian Olteanu, Ioana-Maria Mihai, Florina Bojin, Oana Gavriliuc, Virgil Paunescu

**Affiliations:** 1 Department of Microscopic Morphology, “Victor Babes” University of Medicine and Pharmacy Timisoara, Timisoara, Romania; 2 Clinical Emergency County Hospital “Pius Brinzeu” Timisoara, Center for Gene and Cellular Therapies in the Treatment of Cancer Timisoara – OncoGen, Timisoara, Romania; 3 Department of Functional Sciences, “Victor Babes” University of Medicine and Pharmacy Timisoara, Timisoara, Romania

**Keywords:** Cancer, metastasis, macrophage–cancer fusion cells, cancer fusion cells, CFCs, hypothesis, review

## Abstract

The ability of cancer to adapt renders it one of the most challenging pathologies of all time. It is the most dreaded pathological entity because of its capacity to metastasize to distant sites in the body, and 90% of all cancer-related deaths recorded to date are attributed to metastasis. Currently, three main theories have been proposed to explain the metastatic pathway of cancer: the epithelial–mesenchymal transition (EMT) and mesenchymal–epithelial transition (MET) hypothesis (1), the cancer stem cell hypothesis (2), and the macrophage–cancer cell fusion hybrid hypothesis (3). We propose a new hypothesis, i.e., under the effect of particular biochemical and/or physical stressors, cancer cells can undergo nuclear expulsion with subsequent macrophage engulfment and fusion, with the formation of cancer fusion cells (CFCs). The existence of CFCs, if confirmed, would represent a novel metastatic pathway and a shift in the extant dogma of cancer; consequently, new treatment targets would be available for this adaptive pathology.

## INTRODUCTION

From bacteria to multicellular and complex organisms, cancer can be viewed in many ways as a stand-alone entity that is adaptable and steadfast regarding survival, as observed for any living organism. The nature of cancer is the nature of survival. Cancer acts like virulent and lethal viruses, which eventually kill their host. Approximately 50% of patients diagnosed with cancer are eventually cured with the therapeutic options available currently; however, the remaining 50% of patients ultimately succumb to the disease [1].

In the seminal article entitled “The Hallmarks of Cancer” by Hanahan and Weinberg [2] and in the follow-up revision in “The Next Generation” [3], the authors propose and successfully argue that the complexity of cancers can be explained by rules or more precisely by biological capabilities: sustained proliferative signaling, evasion of growth suppressors, resistance against cell death, immortality, induction and maintenance of angiogenesis, and activation of invasion and metastasis. These hallmarks are joined by emerging hallmarks, such as deregulated cellular energetics and evasion from immunosurveillance/immune-mediated destruction [2,3]. A precise definition of what cancer represents can be given by its apparently limitless drive to replicate.

The scientific research drive to combat cancer has never been more pronounced, with almost 171,000 articles published (PubMed) on this subject in the last 10 years, encompassing all subdivisions of these investigations from fundamental research to clinical trials. Although cancer remains one of the leading causes of deaths worldwide, all these research efforts have been partly effective [4]. Tremendous leaps of knowledge have been made in the understanding of cancer biology [5], the mechanisms of cancer drug resistance [6,7], the associated immunobiology of cancer [8-11], the ability of cancer cell metastasis [12-14], and of course the treatment of cancer [15-20].

However, many unanswered questions remain. There are currently five accepted models of carcinogenesis [21] that try to explain the genesis of a cancer cell. Various studies have confirmed the role played by genes and healthy tissues in the progression of cancer, with the environment surrounding the tumor, i.e., the tumor microenvironment (TME), clearly not remaining an uninvolved participant [22-24]. The ability of a cancer cell to spread to distant sites remains the most important enigma in cancer research that needs to be completely investigated because metastasis causes more than 90% of all cancer-related deaths [12]. Although the inception of the metastatic process is largely well understood by now, many problems need to be addressed, especially in light of the recent advancements in micro- and macro-anatomy uncovered by Benias et al. in the landmark study entitled “Structure and distribution of an unrecognized interstitium in human tissues” [25] and by Louveau et al., who addressed the structural and functional features of lymphatic vessels in the central nervous system, thus paving the way for understanding how cancer metastasizes [26]. Furthermore, the discovery of circulating tumor cells (CTCs) and their ability to seed distant metastasis has given rise to additional avenues of research [27-29] and potential treatment options.

The remaining outstanding enigmas pertain to the ability of a cancer cell or part of it, for example, the nucleus [30], to survive the processes of migration to another part of the body. Regarding this, CTCs have been scientifically known to have a mean diameter of approximately 25 µm and are mostly adherent to platelets (CTCs with aggregated platelets have an even larger diameter), thus hindering their passage through capillary valves, which are approximately 8 µm in diameter. Finally, CTCs face another impediment, i.e., the hydrodynamic shear forces of the circulation system, which would presumably tear the cells apart, making CTCs a very unlikely source of metastasis.

The manner in which cancer cells adapt to the new conditions present in the niche of metastasis and their ability to remain dormant (tumor dormancy) for many years before the secondary tumors are discovered represents some difficult questions that remain to be answered [31-37].

## HYPOTHESES AND REVIEW

We hypothesized that under particular biochemical and psychical circumstances, cancer cell nuclear expulsion [30] coupled with macrophage fusion, which results in a fusion hybrid, is a possible mechanism of survival and metastasis capability of cancer cells.

### Extant prototypes of metastasis

The current scientific view of the models of metastasis has attempted to explain the apparently innate ability of cancer cells to spread to distant sites in the body. Presently, three main theories prevail regarding cancer metastasis: 1) epithelial–mesenchymal transition (EMT) and mesenchymal–epithelial transition (MET) hypothesis; 2) cancer stem cell hypothesis; and 3) macrophage–cancer cell fusion hybrid hypothesis.

#### EMT and MET hypothesis

EMT was first established by developmental biologists as a well-defined program of the cell that performs critical roles in early embryonic morphogenesis [31,32]. The best way to describe EMT is as a trans-differentiating program that is initiated by EMT-inducing transcription factors; thus, EMT provides cells with an epithelial morphology the ability to generate mesenchymal cells. The most important characteristic of EMT is that it is reversible in some contexts; hence, cells that have undergone EMT can revert to the original epithelial state via the reverse program, MET.

**FIGURE 1:**
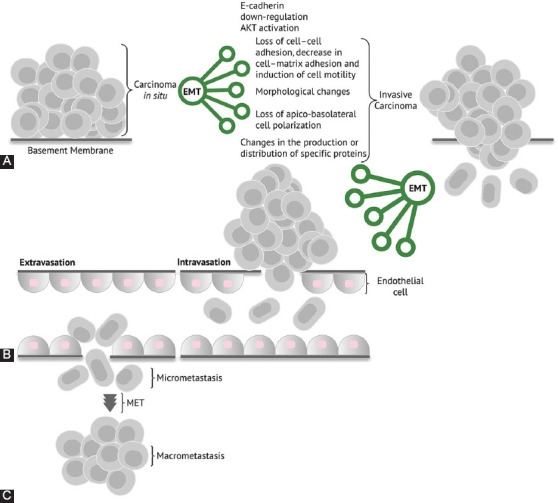
Graphical representation of the epithelial–mesenchymal transition (EMT) and mesenchymal–epithelial transition (MET) hypothesis. (A) Carcinoma *in situ* with established EMT events and characteristics, with ensuing invasive carcinoma. (B) Invasive carcinoma cells with a high migration capability and distant seeding through intravasation and extravasation. (C) Establishment of a metastatic niche with reversal of mesenchymal differentiation via MET.

Numerous studies [33,38-41] have attempted to shed some light on the presence of EMT/MET in cancer cells and its contribution to the ability of cancer cells to metastasize. It is believed that EMT confers stemness (a stem-like state) to cancer cells; thus, stem-like cancer cells would gain the ability to disseminate to distant sites. Important questions remain regarding EMT and its role in cancer metastasis. Often, EMT signals are not detected in many pathological preparations [12,42-44]. The activation of EMT requires the right combination of random gene mutations, gene silencing, or gene augmentation via through epigenetic signaling [45] and the right signaling dynamics between the cancer cells and the TME via contextual signals [22-24]. The eventual seed of dissemination and metastasis needs to undergo the reverse program (MET) and recapitulate its epithelial characteristics at the eventual site of metastasis (Figure 1).

Important questions remain regarding EMT and its role in cancer metastasis. As it stands, the EMT/MET model has credibility in *in vitro* pathways to metastasis alone; therefore, further studies are needed to determine whether EMT/MET is responsible for metastasis *in vivo*.

#### Cancer stem cell hypothesis

Stem cells are known for their ability to proliferate and migrate during tissue morphogenesis and differentiation. In the innumerable cellular niches of the human body, cells can exist in semi-differentiated states, executing the role of tissue renewal. Accordingly, it is assumed that cells that have stem-like characteristics are present among the heterogeneous cancer cell population of a tumor [46].

**FIGURE 2:**
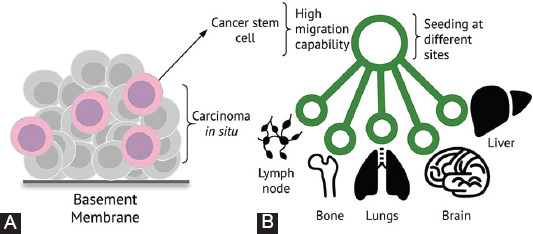
Cancer stem cell hypothesis. (A) Carcinoma *in situ* with cancer cells that possess stem-like features, with basement membrane passage capacity and high through-tissue motility. (B) Seeding of metastatic niches at different sites, with tumor dormancy, which is characteristic of this hypothesis.

Some authors consider these stem cells as the origin of cancer stem cells and metastasis [46,47] (Figure 2). Although many metastatic cancer cells express various characteristics of stem cells or can be considered stem-like counterparts, the expression of these characteristics is not directly proportional to their capacity for distant invasion and metastasis [12,48,49].

#### Macrophage–cancer cell fusion hybrid hypothesis

**FIGURE 3:**
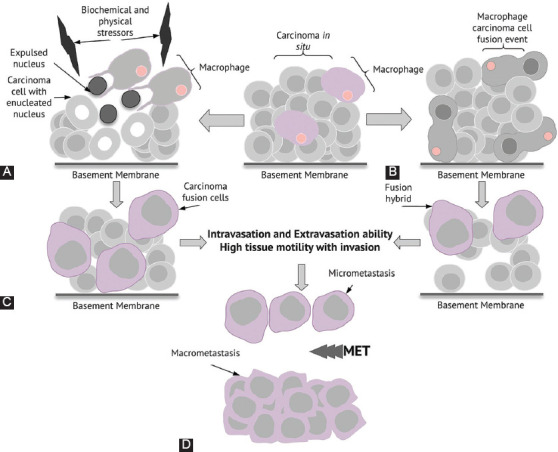
Macrophage–cancer cell fusion hybrid hypothesis and nuclear expulsion followed by the formation of cancer fusion cells (CFCs). (A) Under biochemical and/or physical stress, carcinoma cells can undergo a particular cell-death-escape phenomenon, with expulsion of the nucleus, subsequent engulfment of the expulsed nuclei by tumor-associated macrophages (TAMs), and formation of CFCs. (B) The fusion of TAMs with carcinoma cells and formation of fusion hybrids. (C) Newly formed CFCs and fusion hybrids with high through-tissue motility (characteristic of macrophages) and high seeding capacity without the need for the initial epithelial–mesenchymal transition (EMT) cascade. (D) Metastatic niches established by CFCs and fusion hybrids with mesenchymal–epithelial transition (MET) cascade and the formation of macrometastases.

The roles played by TME [3,22-24] as well as the immune system in the initiation, maintenance, and propagation of cancer are well established [9-12]. Previous studies have reported the role of tumor-associated macrophages (TAMs) as facilitators of tumor development, progression, and metastasis [12,50-53]. Seyfried and Huysentruyt [12] were the first to propose that macrophages or similar cells of myeloid origin are the source of metastatic cells (Figure 3). TAMs can promote the specific expression of CD163 in cancer cells, thereby facilitating metastatic activity [54]. The uniqueness of the proposed hypothesis originates from the fact that cells of the myeloid lineage are already of mesenchymal nature and would not require the complex genetic changes needed for the EMT-to-MET transition. In addition, the fusion of macrophages with epithelial cells in the TME results in fusion hybrids that exhibit the cellular characteristics of macrophages and carcinoma epithelial cells [55,56].

#### Nuclear expulsion and the formation of cancer fusion cells (CFCs)

Based on previously published findings regarding cancer cell metastasis [12,50,53-56], this article aimed to validate the notion of cancer cell nuclear expulsion [30] coupled with macrophage fusion resulting in the formation of CFCs, with a high migration capacity, distant seeding, and macrometastasis formation (Figure 3). To expand our proposed hypothesis, several factors should be addressed or explained. Under well-documented physiological conditions [57], nuclear expulsion is encountered in erythroblastic islands formed between macrophages and erythroblasts in tissue niches that support erythropoiesis. Erythroblastic islands are essential for adequate erythropoiesis. Erythroblast macrophage protein (Emp), which is a key protein that is expressed on macrophages and erythroblasts, plays an important role in nuclear expulsion. Moreover, the absence or loss of function of Emp in the erythroblast population inhibits nuclear expulsion [58]. If Emp is expressed *de novo* on cancer cells, likely because of an increase in dedifferentiation that leads to a more embryonic-like phenotype, Emp or other proteins with a similar function might represent a mechanism of cancer cell nuclear expulsion. Hence, the study of Emp is a plausible research avenue for the validation of our hypothesis.

Another area of future research is the investigation of the aspect of nuclear integrity. Several molecules, such as phosphoinositide 3-kinase beta (PI3Kβ), which regulates the nuclear envelope (NE) through upstream control of regulator of chromosome condensation (RCC1) and RAs-related nuclear protein (Ran) activity, contribute to the stability of NE [59]. PI3Kβ is known to be overexpressed in many carcinomas [60]; thus, it is logically fitting that the nuclei of cancer cells would have very stable NEs and that the extruded cancer cell nuclei would retain their nuclear integrity.

Next, the engulfment of the expulsed cancer cell nuclei by macrophages, followed by the formation of CFCs, needs to be addressed. Macrophages are well-established cellular components of phagocytosis that possess two main pathways for cellular clearing: efferocytosis and antibody-dependent cell phagocytosis (ADCP). The CD45 transmembrane protein is a principal component of the negative feedback signal in both efferocytosis and ADCP. CD45 functions as a “do not eat me” signal when it couples with SIRPα receptors on macrophages [61]. Expulsed cancer cell nuclei no longer express CD45; therefore, there is no negative feedback signals for macrophage engulfment. In addition, a special type of cellular engulfment has been shown to be present in cancer, in which the existence of a “cell-in-a-cell” feature is often identified. This feature, which is termed entosis, represents a non-apoptotic cell death pathway, wherein a cancer cell is engulfed by another cancer cell, i.e., cell-in-cell invasion [62]. The cell-in-cell invasion evolves over three steps: degradation by lysosomal enzymes, release of the cell, and fusion of the cells. The fusion ability of cancer cells can be considered the bedrock of all metastatic pathways, with offshoots and components of the fusion of cells being present in all of them [63-65]. Accordingly, entosis of cancer cell nuclei by macrophages, with secondary fusion between the nucleus of the macrophage and the engulfed nucleus, would represent a feasible conjecture or explanation for the formation of CFCs. The newly established cells would express molecular signatures of contributors of both lineages.

## TESTING THE HYPOTHESIS

To test and validate our hypothesis, several steps are needed and a confirmation trial of error study and a validation “tree” are required. This base-to-stem and expansion approach requires scientific answers to certain questions. Cancer cell enucleation [30] needs to be further confirmed on multiple cancer cell lines. In addition, cancer cell nuclear viability (i.e., the maintenance of nuclear integrity and stability of genetic information) after expulsion requires multi-tiered validation. To validate the hypothesis of CFCs, first, the mechanism underlying the fusion between TAMs and the expulsed cancer cell nuclei has to be examined. This can be achieved by the establishment of several TAM cell lines seeded with captured cancer cell nuclei, followed by the examination (e.g., molecular markers and whole-genome sequencing) of the presence of CFCs. Cancer cell nuclei can be harvested using extant techniques that are used for the isolation and enrichment of CTCs [66]. Subsequently, after the confirmation of the presence of CFCs, various preponderant cancer cell characteristics, such as deformability, density gradient, cell polarity, electrical charge, epithelial cell adhesion molecules, cytokeratins, and expression of tumor-associated markers, should be verified. Finally, the ability of CFCs to establish macrometastases *in vitro* and *in vivo* will be a fundamental validation step for our hypothesis.

## MEDICAL IMPLICATIONS

Our proposed hypothesis would have various medical implications, particularly regarding the process of identification of a new target for cancer therapy. The targeting of TAMs and CFCs will create a new avenue in the research of tumor and metastasis treatment, thus potentially providing new therapies and the possibility of cessation of cancer metastasis, which would transform cancer into a chronic and manageable disease.

If confirmed, our hypothesis will lead to debulking of the burden of cancer that weighs down the global health system and social system as a whole [67].

## CONCLUSION

Cancer as a distinct biological entity needs to be viewed in the clear light of adaptable evolution to existing changes. The presence of multiple lines of survival for a cancer cell represents a distinct and factual argument. The confirmation of the existence of CFCs as a distinctive pathway for human cancer metastasis will generate positive medical avenues for the entire global social and healthcare system.
